# Atrial Septal Defect in a Very Old Woman

**DOI:** 10.4021/cr254e

**Published:** 2013-03-08

**Authors:** Elika Pinho, Andre Amaral Gomes, Maria Joao Silva, Tiago Pinheiro Torres, Andreia Coelho, Pedro Bernardo Almeida, Patricia Lourenco, Paulo Bettencourt

**Affiliations:** a Servico de Medicina Interna, Centro Hospitalar Sao Joao, Portugal; b Servico de Cardiologia, Centro Hospitalar Sao Joao, Portugal; c Unidade I&D Cardiovascular do Porto, Faculdade de Medicina da Universidade do Porto, Portugal

**Keywords:** Atrial Septal Defect, Congenital heart diseases, Diuretic therapy

## Abstract

Atrial Septal Defect (ASD) is one of the most frequently congenital heart diseases in adults and it is often asymptomatic until adulthood. We report a case of a 90-year-old woman admitted to hospital with dyspnea and orthopnea insidiously progressing over the preceding 5 years and becoming severe with dyspnea on minimal activities, orthopnea and paroxysmal nocturnal dyspnea, in the last 2 weeks. The transthoracic echocardiogram revealed an atrial septal defect ostium secundum type, with left-to-right shunt, moderate to severe tricuspid insufficiency, severe pulmonary hypertension (72 mmHg) and preserved biventricular function. With diuretic therapy optimization the patient showed symptomatic improvement. This present case represents and unusual and very late presentation of an atrial septal defect ostium secundum type, which is usually diagnosed at the mild adult age. Our patient lived symptom-free for over 80 years.

## Introduction

Atrial Septal Defects (ASD) accounts for 25-30% of the newly diagnosed congenital heart defects in adults [1]. Although most of ASD result from spontaneous genetic mutations, some are inherited [2]. Anatomically, it is characterized by an open communication in the interatrial septum with a left to right shunt, resulting in variable right cardiac chambers dilatation depending on the size of the shunt and ventricular compliance. ASD are classified according to their location and nature of the embryologic defect. Ostium secundum is a communication in the fossa ovalis and makes up 60-75% of all ASD cases. Other types of ASD are ostium primum type when the defect is in the lower part of the atrial septum and sinus venous type when the communication is in the upper atrial septum [1-3]. ASD remain frequently underdiagnosed or late diagnosed because symptoms are initially absent or indolent and usually take three to four decades to require surgical correction [4, 5]. Potential complications of an undetected atrial septal defect include right ventricular failure, paradoxical embolization, atrial arrhythmias and pulmonary hypertension that may become irreversible and leads to right-to-left shunting (Eisenmenger Syndrome) [2, 5].

## Case Report

A 92-year-old Caucasian woman was admitted in the emergency department with dyspnea at rest, pleuritic chest pain and palpitations, symptoms that had started suddenly 3 hours before. She had no cough and fever and no constitutional symptoms. She had a 5-year history of exertion dyspnea that had progressed to dyspnea on minimal activities, orthopnea and paroxysmal nocturnal dyspnea in the previous two weeks.

She had past history of epilepsy, type 2 diabetes mellitus, hypertension, peripheral arterial disease with a left femoropopliteal bypass surgery 3 years before.

Her regular medications included phenobarbital (200 mg daily), glibenclamide (5 mg daily), lisinopril (20 mg daily), acetilsalicilic acid (100 mg daily) and clopidogrel (75 mg daily).

In the emergency department, she had no fever, the blood pressure was 156/86 mmHg, the pulse was irregular and the heart rate was 120 beats per minute, the respiratory rate was 24 breaths per minute and SaO2 (fraction of inspired oxygen (FiO2)) of 96% while breathing ambient air. She had crackles in both inferior pulmonary half-fields, and an III/VI holosystolic murmur was audible at the upper left sternal border. Jugular venous pressure was elevated and she had hepatic enlargement, with the liver margin palpable 5 cm below the costal rib; lower limb edema was exuberant and till above the knees.

The complete blood count was normal and C-reactive protein level was negative. Her plasma creatinine was 1.4 mg/dL and the urea was 107 mg/dL; plasma electrolytes and urine analysis were normal. Troponin I and CK-MB are normal with an elevated BNP, 2501.2 pg/mL.

The electrocardiogram showed sinus tachycardia (120 beats per minute), right axis deviation and incomplete right-bundle-branch block, but there were no signs of ischemia. Chest X–ray showed enlarged heart silhouette and hilar engorgement.

**Figure 1:**
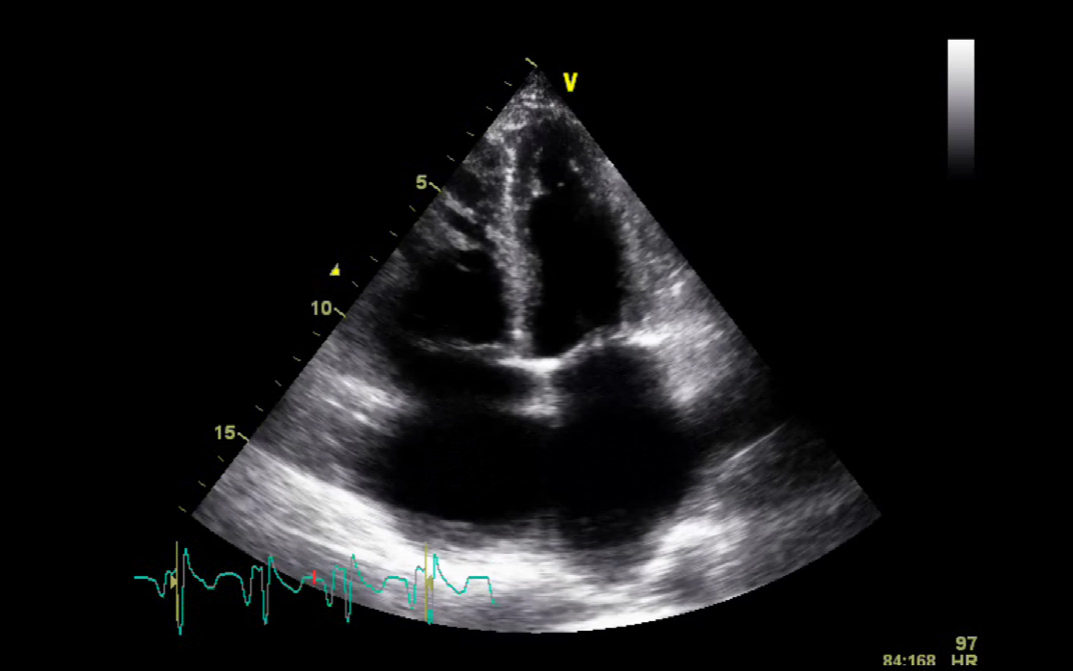
Dilatated right cardiac chambers and left atrium in four chamber apical view.

**Figure 2:**
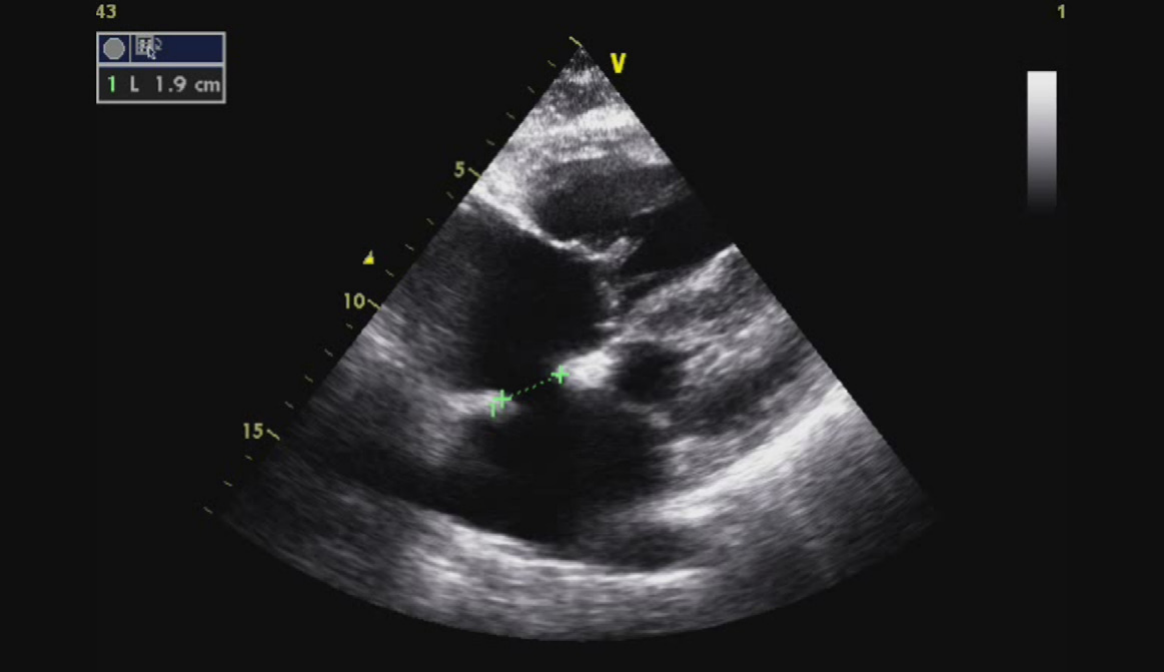
1.9 cm ostium secundum type atrial septal defect in subcostal view.

**Figure 3:**
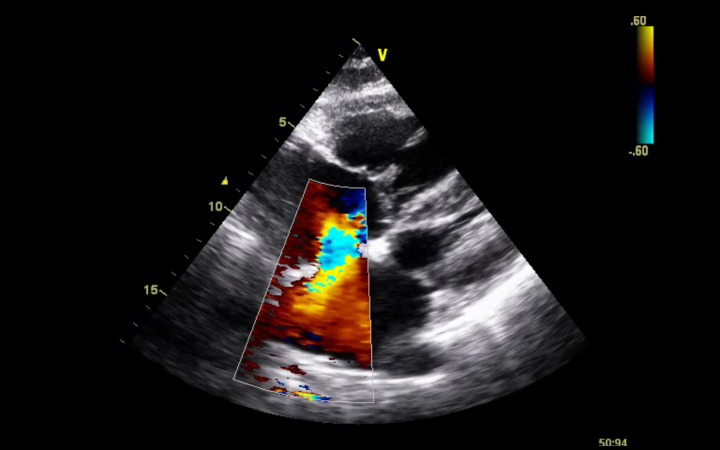
Ostium secundum type atrial septal defect with a left-to-right shunt subcostal view.

The patient was admitted in the Internal Medicine ward and a transthoracic echocardiogram was performed. She had dilated right cardiac chambers and left atrium (Fig. 1), an atrial septal defect ostium secundum type of 1.9 cm and a left-to-right shunt was documented (Fig. 2, 3). A tricuspid regurgitation was also evident with an estimated pulmonary artery sytolic pressure (PASP) of 74 mmHg. Moderate atrial regurgitation was also documented. The ventricular function of both ventricles was normal.

The patient initiated intravenous therapy with loop diuretic with excellent diuretic response, no additional renal function deterioration and successful control of her hypervolemia. She was discharged five days later, in New York Heart Association classe II, and remained clinically stable.

She died 1-year latter for uncontrolled epilepsy in an admission due to a lower limb cellulitis.

## Discussion

Atrial septal defect (ASD) is among the most common congenital heart diseases in adults and it occurs in women two to three times more often as in men [1, 2]. Fossa ovalis defects are classified as ostium secundum and are the most common type of all ASD. The remaining types include ostium primum (15% of all cases) and sinus venosus (10% of all cases) [1].

Regardless of the anatomic location, the pathophysiologic consequences of an ASD result from the shunting of blood from one atrium to the other. The shunt direction and its magnitude are determined by the size of the defect and the relative compliance of the ventricles. Patients are usually asymptomatic when the defect is associated with a small shunt and no hemodynamic consequences are expected. However, when a sizable defect associates with a large shunt and important hemodynamic impact, there will be right chambers and pulmonary arteries enlargement and this may culminate in the development of right ventricular failure and shunt reversing [1, 2].

ASD remain undetected for years; symptoms usually take 30 - 40 years to develop and they appear insidiously since right sided volume overload is well tolerated. The diagnosis of ASD is made when substantial left-to-right shunt occurs (a pulmonary to systemic flow of 1.5). Over the years, the increased volume of blood flowing through the right heart chambers causes right ventricular dilatation and ultimately failure. Rarely Eisenmenger’s Syndrome may develop [1, 2, 5].

By the age of 40 years old, 90% of the untreated patients developed a typical clinical presentation with exertion dyspnea, fatigue and palpitations. Life expectancy of unrepaired ASD is generally shortened with observational reports of less of 50 percent of these patients surviving beyond the age of 40 and only 10 percent reach the age of 60 [6].

Echocardiography is the imaging modality of choice for the diagnosis of ASD. Evaluation shall include demonstration of shunting across the defect, assessment of right ventricle volume overload and evaluation of any associated anomalies [7]. Transthoracic echocardiogram has high sensitivity in the diagnosis of ostium primum and ostium secundum defects, but sometimes does not identify sinus venous type defect. The sensitivity of this modality may be enhanced by contrast echocardiography. Transesophageal and doppler color-flow echocardiography can also provide information in ASD patients; they are useful in detecting and determining the ASD location and in identifying sinus venous defects and anomalous pulmonary drainage. Echocardiography may provide hemodynamic information to estimate shunt flow through the defect and to noninvasively determine pulmonary artery pressure in most patients. In selected patients, cardiac catheterization may be required to determine the exact magnitude and direction of the shunt, as well as to evaluate and quantify pulmonar hypertension [7].

The optimal treatment of ASD is surgical or percutaneous transcatheter closure. An ASD with a pulmonary to systemic flow ratio of ≥ 1.5 should be repair to prevent right ventricular dysfunction [6, 7].

Surgical closure in elderly patients with pulmonary hypertension, congestive heart failure or arrhythmias is controversial [6, 8, 9]. Patients with severe pulmonary hypertension, like our nonagenarian patient, are not candidates for surgical repair.

Our ASD patient remained asymptomatic until over 80 years of age, when she initiated signs and symptoms of right heart failure and congestion. This is a case of a rare late evolution and presentation of an ASD ostium secundum type. Considering the patients’ very old age and the severity of her pulmonary hypertension a conservative approach was decided. She died 1-year latter for an unrelated cause.
